# Recognizing Brain Tumors Using Adaptive Noise Filtering and Statistical Features

**DOI:** 10.3390/diagnostics13081451

**Published:** 2023-04-17

**Authors:** Mehwish Rasheed, Muhammad Waseem Iqbal, Arfan Jaffar, Muhammad Usman Ashraf, Khalid Ali Almarhabi, Ahmed Mohammed Alghamdi, Adel A. Bahaddad

**Affiliations:** 1 Department of Computer Science, Superior University, Lahore 54000, Pakistan; 2 Department of Software Engineering, Superior University, Lahore 54000, Pakistan; 3 Department of Computer Science, GC Women University, Sialkot 51310, Pakistan; 4 Department of Computer Science, College of Computing in Al-Qunfudah, Umm Al-Qura University, Makkah 24381, Saudi Arabia; 5 Department of Software Engineering, College of Computer Science and Engineering, University of Jeddah, Jeddah 21493, Saudi Arabia; 6 Department of Information System, King Abdulaziz University, Jeddah 21589, Saudi Arabia

**Keywords:** brain tumor, magnetic resonance imaging, contrast stretched enhancement, anisotropic, filtration, segmentation, classification, morphological operation

## Abstract

The human brain, primarily composed of white blood cells, is centered on the neurological system. Incorrectly positioned cells in the immune system, blood vessels, endocrine, glial, axon, and other cancer-causing tissues, can assemble to create a brain tumor. It is currently impossible to find cancer physically and make a diagnosis. The tumor can be found and recognized using the MRI-programmed division method. It takes a powerful segmentation technique to produce accurate output. This study examines a brain MRI scan and uses a technique to obtain a more precise image of the tumor-affected area. The critical aspects of the proposed method are the utilization of noisy MRI brain images, anisotropic noise removal filtering, segmentation with an SVM classifier, and isolation of the adjacent region from the normal morphological processes. Accurate brain MRI imaging is the primary goal of this strategy. The divided section of the cancer is placed on the actual image of a particular culture, but that is by no means the last step. The tumor is located by categorizing the pixel brightness in the filtered image. According to test findings, the SVM could partition data with 98% accuracy.

## 1. Introduction

Medical image processing is a most challenging and in-demand field. Magnetic resonance imaging (MRI) has emerged as a dynamic area of medical image processing for detecting brain tumors [1]. One of the most challenging processes in image processing, image segmentation, determines how accurately the results will turn out. Image segmentation is a process of dividing an image into distinct sections.

Segmentation utilizes a library of MRI-scanned images. MATLAB software determines the classification and identification of cancer from brain MRI images. A tumor is a tissue mass that develops in a disorganized way that normalizes development. Due to its persistent, severe, and infiltrative nature, a brain tumor is intrinsically serious and essential [2]. It is easy to recognize the margin of a benign brain tumor periphery. These cells should not invade the tissue around them or bond to other body areas, but they can still compress the brain’s receptive region and cause severe health problems.

Cancerous cells are found in malignant brain tumors. They are more likely to develop rapidly and invade; very rarely, cancerous cells in the surrounding healthy brain tissues may break free from a malignant brain tumor and spread to other parts of the body. The spread of cancer to other areas of the body is called metastasis. Imaging plays a key role in diagnosing a brain tumor, and a neurological examination typically includes identifying a brain tumor. Doctors use the medical information to categorize the tumor from the least insistent to the most insistent, allowing doctors to verify the most effective course of care. MRI (magnetic resonance imaging) is one of the most widely preferred diagnostic techniques [3]. MRI is a special medical imaging tool that produces high-quality human body images with high spatial resolution and considerable soft tissue discrimination. Anatomical knowledge is used to analyze the development of the human brain and assess anomalies.

Several segmentation strategies exist based on similarity or discontinuity, such as threshold and area-rising approaches. The machine learning methodology has now strengthened these approaches. Primary and secondary brain tumors are divided into different categories since primary brain tumors develop in the brain itself, such as astrocytomas (AS), glioblastomas multiform (GBM), and meningiomas (MEN), and secondary brain tumors are cancer cells that first develop in another part of the body and then spread to the brain. Healthcare professionals may now serve patients with high-quality healthcare thanks to the integration of information technology and e-health care systems in the medical industry. This study uses the classifier function extraction technique and support vector machines to distinguish aberrant brain tissues from normal brain tissues such as grey matter (GM), white matter (WM), and cerebrospinal fluid (CSF) in magnetic resonance (MR) images. A brain tumor is essentially an uncontrolled growth of brain cancer cells, whereas a tumor is an unchecked proliferation of cancer cells in any body part. Brain tumors can be benign or cancerous. While malignant brain tumors are physically heterogeneous and contain active cells, benign brain tumors are architecturally homogeneous and lack active cells. Gliomas and meningiomas are examples of low-grade tumors classified as benign tumors, and glioblastoma and astrocytomas are subsets of high-grade tumors classified as malignant tumors. Segmentation is used to distinguish contaminated tumor tissues from medical imaging modalities [3]. Segmenting an image into distinct areas or blocks with comparable and common characteristics, such as color, texture, contrast, transparency, boundaries, and degree of grey is crucial in image analysis. Brain tumor segmentation separates solid tumors such as WM, GM, and CSF from normal brain tissues and edematous tumors like WM, GM, and edema using MR images or other imaging modalities.

Abnormal images from one MRI scan. MRI is another name for the multimodality of the MRI [1]. To effectively classify the tumor location, MRI enables the monitoring of multiple RMI sequences such as T1, T2, and T1c, which reveal the complete brain anatomy. MRI is helpful in modeling and creating an aberrant brain atlas of the general pathological brain [2]. Region-based and machine-learning approaches are employed in portions of more than one RIM modality [3].

In the immediate area, the radiologist measures the tumor’s scale, location, and effect when a brain tumor is medically detected. With proper tumor-range detection at the initial stage [4], tumor patients can significantly increase their odds of survival. Standard division methods are lacking in cases of a brain tumor [5] where the cancer is close to where brain tumors are found. Current work needs considerable improvement to increase tumor detection accuracy, applicability, and automation [6]. The moderate adaptive classification template [7] and the maximized expectation (EM) [8] are statistical methods that fail when there are multiple brain region deformations. Different MR images are used to obtain final segmentation results to solve the problem, such as T2 and T1, as well as enhanced contrast, PD, and geometric constraints [9].

Malignant and benign are two designated forms of cancer. There are fast-developing and cancerous malignant tumors. Benign tumors grow slowly and are non-cancerous and less toxic than malignant ones. Electron microscopy is the method of producing a visual image for the therapeutic research of the human body, and it is adequate for detecting the probabilities of non-invasion. Different non-invasive medical imaging technology forms include X-ray, CT scan, SPECT, MRI, PET, and ultrasound [10].

This study focuses on identifying brain tumors through methods of image processing. In comparison to other diagnostic imaging approaches, magnetic resonance imaging (MRI) is commonly utilized as it gives better contrast and more detailed images of cancerous tissues. Therefore, brain tumor detection can be achieved via MRI images. Research has focused on the texture and statistical information of images to train the support vector machine (SVM). Thus, better performance among state-of-the-art methods can be achieved which would allow tumors to be classified more accurately.

The tumor tries to squeeze the surrounding cells, and tension is emitted. Moreover, this also happens as the tumor restricts the fluid that circulates in the brain. The common symptoms are vomiting, headache, nausea, and issues with balancing and movement. Brain tumors can be identified by medical imaging modalities such as MRI and CT scans. Both modalities have recognition benefits, depending entirely on the position, type, and purpose of the appropriate exam. In this study, we use MRI images even though they are easy to interpret and provide accurate classification and foreign mass location.

Providing resources for clinical diagnosis is the key reason for identifying brain tumors. The study goal is to accurately classify tumors using brain MRI images. The goal is to provide a tumor presence algorithm by combining multiple procedures to provide a foolproof algorithm for detecting if there is a tumor in each MRI Image, and if there is, to specify the tumor’s location with highly accurate results.

Three phases can be used to describe the proposed structure. The first phase involves a filtration method that reduces noise using the anisotropic filter (A.F.) of the brain MRI image, followed by adjustment-based segmentation that uses a structuring element to segment the tumor area from the filtered images. A morphological process that identifies the tumor’s position in the original image is part of the third stage.

## 2. Literature Review

Brain tumor detection through image processing is an important topic nowadays. Nowadays, MRI is an essential technique used in the medical field. It can detect the damaged part of the body that consists of a brain tumor. Using MRI techniques can also help to see the actual size and shape of a brain tumor. One of several strategies used to determine brain cancer specifications is the adaptive curved area curvature (ACAC) technique. A support vector machine (SVM) is used to classify a normal or anomalous case of a brain hemorrhage. When the SVM collects the result of an abnormal sense of brain tumor at that time, the abnormal condition is maintained throughout the segmentation process. The human body naturally has 3D complicated structure mechanisms, but MRI works only on 2D images. The exact shape of a brain tumor in 2D is not precise. So, 3D is more reliable and helpful for doctors because it can help during an operation. Image matching throughout rapid mode is used to build the 3D model. The 3D model is used to identify the original volume of a tumor in the brain [11]. When MRI images are collected, they consist of lots of noise that affects the accuracy of the images. Many methodologies for filtering are used to reduce the noise of photos. The two mean and median filtering methods are Gaussian minimum and maximum filter. These filters, when combined, decreased the noise of MRI images [12]. The filter of the median is highly utilized for reducing the noise, while the mean filter is used for the low-level noise [13]. A Wiener filter is used to improve image resolution [14]. It proposed an alternative median filter adaptive for enhancing the image [15]. To detect the image boundary, the convex active contour model is used. When explored the issue of tumors in the brain, they removed images from MRI scans utilizing MATLAB software.

The authors used the contour of adaptive convex area algorithm to identify brain tumors. They collected forty sets of images containing brain tumors, and then selected the main feature of the image from the data gathered. They performed the feature extraction of the brain tumors through the cooccurrence matrix of the grey degree algorithm. The MRI images were identified by the support vector machines (SVM), and even the adaptive convex area contour (ACRC) methodology was used to show whether the brain tumor is indeed healthy or abnormal. After that, they implemented the image matching (RMIM) rapid mode method for converting these 2D images into 3D images because the image set collected through MRI is in the 2D form and also uses the cubic interpolation approach. When the images are converted into 3D, the volume (density) of the tumor in the brain is detected. Measuring the importance of a brain tumor is a challenging task. For this, the cubic bounding approach is used. The ellipsoid, sphere, and cylinder are three distinct forms.

The authors suggested an approach to identify a tumor from such an MR image of the brain. Utilizing racially discriminatory cluster analysis, they use superpixel scale zones of the brain’s MR image and total clustering. They operate the HAAR wavelet transform to extract features, including its MR image. Because of such a classification model, AdaBoost was associated with a random forest classifier for generating a classifier because the brain area is classified based on whether it would be usual or unusual, and the image attribute is retrieved by transforming the HAAR wavelet. This approach delivers 100% accuracy on the Brain Web Dataset. They used the MATLAB tool for this study [16].

In the biomedical imaging field, superpixels have become readily available. Superpixels have the power to monitor localized image characteristics but also display consistency, therefore reducing computational issues. In simple terms, it is open and helpful in dealing with problems such as high frame rates, low-quality segmentation, inaccurate volume, as well as structure. Superpixels are being used in the health world for some of these sorts of application fields, such as image analysis segmentation algorithms and skeletal structure approximation, as required [17]. HAAR wavelet transform provides the facility of MR photodecomposition by time horizon visualization, which is very useful in the classification phase. The wavelet transforms only for the extraction of properties pass the two filters through image one, a filter with a low pass and a filter with a high pass. When the data of the image has passed in both filters it extracts a different signal. The signal of outcome for the high pass filter is applied to another pair of filters [18]. ADBRF is a combination of two algorithms. ADB implements the classification in a very easy way: the nature of this algorithm is non-parametric because it integrates the program with another classifier. RF is an algorithm for machine learning; it has been useful for removing noise and evaluating missing data. This algorithm is run on a large dataset and evaluates the main feature for classification. This algorithm is used for binary classification in 0 and 1 form; 0 is used for the normal and 1 is to show the abnormal condition of a brain tumor [19].

The correct and automatic classification of MRI images is increasingly important in the medical area. The authors detected brain tumors using different discrete wavelet transform (DWT) but also continuous wavelet transforms (CWT) as well as support vector machine (SVM) tools. Their methodology consists of three layers in which they define all the detection processes of a brain tumor, as well as preprocessing in which they use the wavelet transform with thresholds for removing the noise and other image problems. They used HAAR, SYMLET, MORLET, and Daubechies techniques for removing the noise of MRI images. HAAR transform is the easy form, and it has a discontinuous nature. It works stepwise and it is used to analyze the image resolution correctly. Performance assessment and survey are accomplished using signal and peak signal-to-noise ratio (PSNR), as well as mean square error (MSE). Daubechies transform guide to the wavelet-based foundation for transform on multidimensional signal refinement development. MORLET or SYMLET have a symmetrical form as well as no function for scaling [20]. The authors trained a support vector machine for image training. Then they gave the new MRI images to the trained SVM for output. They used SVM, DWT, and CWT for brain detection work. In their work, they observed that the CWT is the best wavelet transform that produces an accurate detection of a brain tumor. The CWT loses the edge of the image in segmentation. CWT gives a higher computation result than DWT. If we want to get better visualization, such as matching feature detection, it is best to use CWT. DWT is best for de-noising, compression, and restoration. In their study, they used two different types of datasets for the classification of the support vector machine (SVM) function. Using a support vector machine, they got the best hyper-lane that detects all the points about the problem of one dataset from those of other datasets. For classification, they trained the dataset using kernel and linear classifiers. When they trained the data, they got the best result through the linear classifier. In their research, the use of kernel classifiers is waste of time and not suitable for memory.

The authors used an MRI scanner machine identification of even a tumor. Their method for the identification of the tumor of the brain consists of a median filter, threshold, largest contour, watershed segmentation, and crop. In their study, all the MRI image scans are executed in an axial view. The size of MRI images is 512 × 512 pixels. They use a median filter for the removal of noise and detection of an edge (corner) of the image. A watershed approach is used only for transferring the MRI image into the grayscale and distributing a gray image. The main use of watersheds is to eliminate the skull bone area in the brain. Sometimes the image size is larger, and it is also hard to identify the very same tumor region for this problem as the cropping technique is used. The cropping technique cuts the skull bone area and finds the exact area of the tumor. To change the background of the image, the threshold approach is used. The threshold switches the background color to black but also lets the object shift its shade to white. A binary threshold is just the threshold category. When the pixel intensity becomes greater than the threshold value, then just the pixel value gets retrieved by that of the highest value (the threshold also has intensity values). The pixel intensity produces zero or blacks when this thresholding has a grey intensity value that is lesser than that of the threshold at a certain moment. To calculate the outcome of the brain tumor, the algorithm of the largest contour is used. First, load the threshold image, and then the threshold converts the image into a grayscale; then, stock all of the shapes as vectors; after that, find the largest shape and get the result. To get the biggest contour thresholding result it is necessary to execute this repeatedly. The separate contours contain segmentation performed in cancers, and several other regions are segmented in this tumor, including certain neural tissue, neural serum, including flat discovery. In short, their study shows the result of the brain tumor region and brain region. They calculate the brain tumor region using a mathematical formula. They calculate the region of brain tissue through the number of pixels of the brain [21].

Today, the detection and classification of brain tumors is a common problem. The study suggests a methodology that detects a brain tumor from a given MRI image. His methodology consists of image production, enhancement, segmentation, identification of features, choice of features, as well as classification. BraTS datasets that consist of MRI images were used for this purpose. The MRI images consist of a lot of noise that creates a problem in detecting the tumor. For the solution to this problem, a lab color transform is combined with principal component analysis (PCA). The lab color device is unconventional, and it has bigger dimensions than other color transformations. In the LAB word, the L word defines the value that lies between black (0) and white (1). The A word defines the color scheme from green to red, and B defines the color scheme from blue to yellow. On the other hand, the principal component analysis (PCA) decreases the image problems that are in group form. To boost or improve the image quality, contrast-limited adaptive equalization (CLAHE) is used. It is one of the algorithms that is most used in the medical image processing field. Tumor segmentation of the MRI images involves skull transfer and multi-level thresholding on object identification, and the application of the Chan–Vese algorithm. To compute the MRI image contrast or enhancement, the OTSU threshold approach is used as opposed to the MRI images being changed into the binary form. The OTSU method is used again to remove the skull image into a binary image. This process is repeated at least three times, and in the end the exact excepted region of the tumor is identified. After that, the Chan–Vese algorithm is used to identify the last segmented object. It is a contour model used for segmentation. The author uses a genetic approach for selecting features. For categorization, an artificial neural network is used. This classifier is used to get and train the feature from the feature selection section and show the brain tumor area size and type of tumor. The accuracy of his approach is 95.5% through BraTS [20].

The outcome is distinguished using ANFIS, backpropagation, then the K-NN classification algorithm of the Berkeley wavelet transform (BWT) as well as support vector machine (classifier) classifier just in support, displaying measurements such as specificity, accuracy, and efficiency. The authors achieve 96.51% accuracy through the given technique [22].

The study introduced a new model that is a convolutional neural network named BrainMRNet. That new design included an unused chain and has been based on attn configurations as well as a hypercolumn approach. They preprocessed images in BrainMRNet. After preprocessing an image, the image is sent to the attention module using the image augmentation approach. The attention module selects all the necessary regions of the image after choosing the image that is sent to convolutional layers.

The BrainMRNet model is used on Python libraries. However, the classification results obtained from the pre-trained convolutional neural network (CNN) were AlexNet 86.93%, GoogleNet 89.66%, and VGG-16 84.84%. The Brain MR Net model was however only given training on this data collection. Using this model, 96.0% sensitivity, 96.08% specificity, and 96.05 percent correctness were obtained. The final accuracy of this brain tumor detection method is 96.05%. In their study, the authors merged normal and abnormal MR images. They also used the Naïve Bayes approach for new image classification and obtained 84.17% accuracy [11].

Most of the research focuses on predicting tumors using MRI-based datasets, which are publicly available from different repositories. Existing research has not focused on preprocessing an image as an MRI image may contain noise due to instrument failure, so it can be covered before extracting features. Before extracting features, noise removal makes the image clearer, and statistical features can differentiate segments efficiently. Recent studies have focused on textural elements that can be large in number. Thus, statistical features using Gray-Level Co-Occurrence Matrix (GLCM) are being used in this research which is a statistical technique for feature extraction. SVM is a widely used classifier and is being used for binary classification for this problem.

## 3. Research Methodology

**Figure 1:**
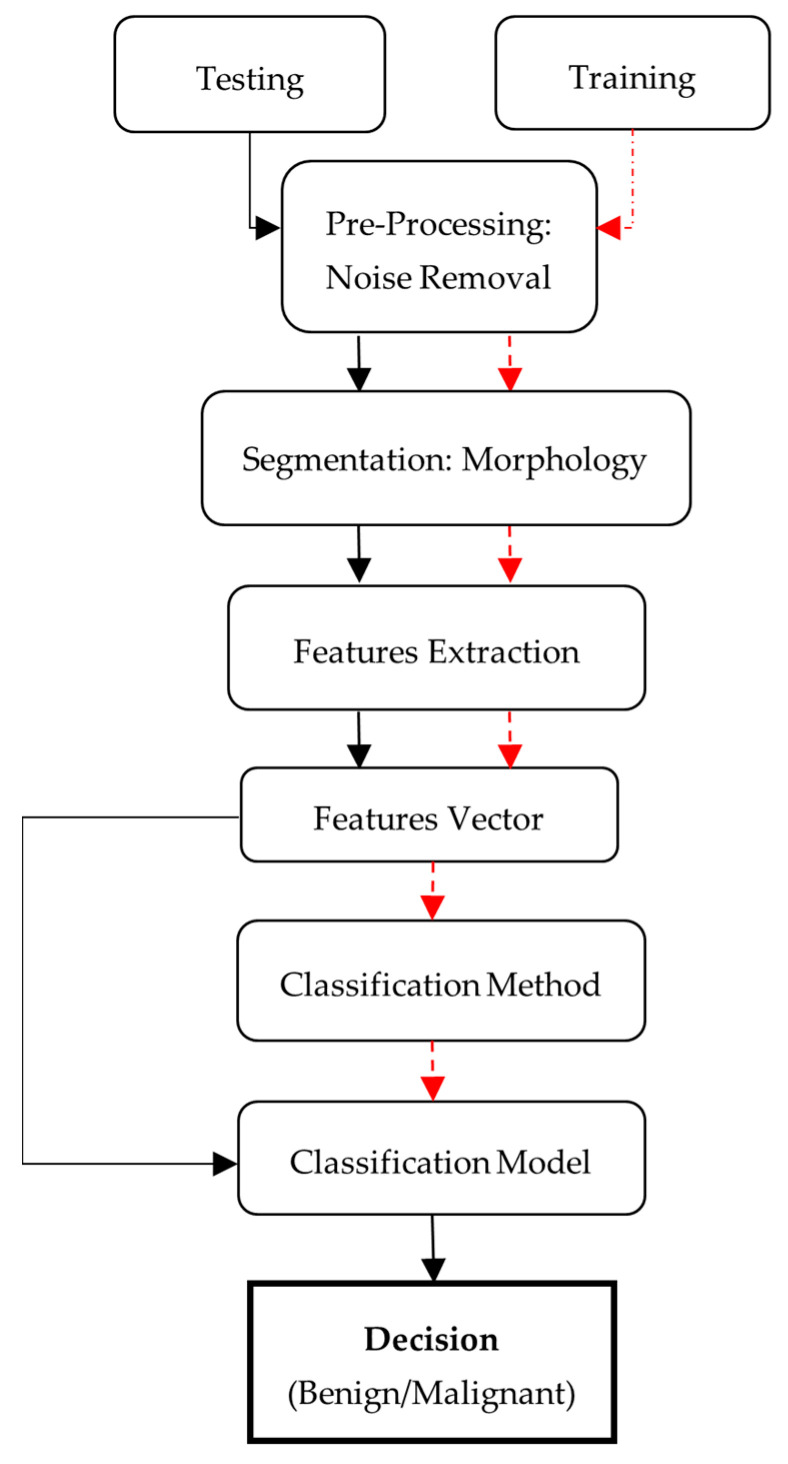
Proposed framework for MRI brain tumor classification.

The nature of the proposed strategy is shown in Figure 1. Initially, there is a need for MRI images, which is also the first step of the proposed methodology, known as image acquisition. For this purpose, datasets that contain human brain MRI images have been used. After this, input image pre-processing is applied followed by post-processing. There are different steps to be performed. The proposed method can be represented in the following steps:

- Noise removal from MRI image using adaptive filtering method.
- Statistical feature extraction using GLCM.
- Classification of benign and malignant tumors using SVM by features extracted.

### 3.1. Acquisition of an Image

**Figure 2:**
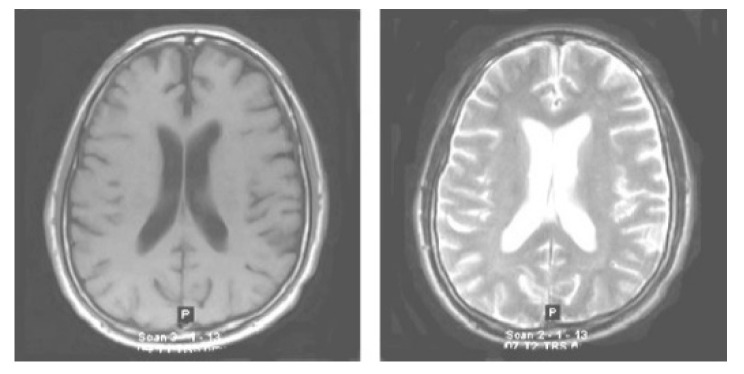
Original normal brain T1-weighted and T2-weighted MRI images.

The very first stage of the suggested methodology is the acquisition of an image, in which an MRI image is used as an input for processing. In the application of the suggested methodology, brain tumor MRI images have been used. Four datasets containing T1-w and T2-w contrast-enhanced MRI images of three kinds of brain tumors were used. Images in the MRI format are stored in MATLAB R2021a, 255 × 255 in size, and they are shown as RGB images of grayscale as the ranges of their entries are from 0 to 1. Here, the black image is represented by 0 while the white image is represented by 1. Entries between 0 to 1 represent the variation in intensity from black to white (see Figure 2).

### 3.2. Datasets

#### 3.2.1. BraTS

**Figure 3:**
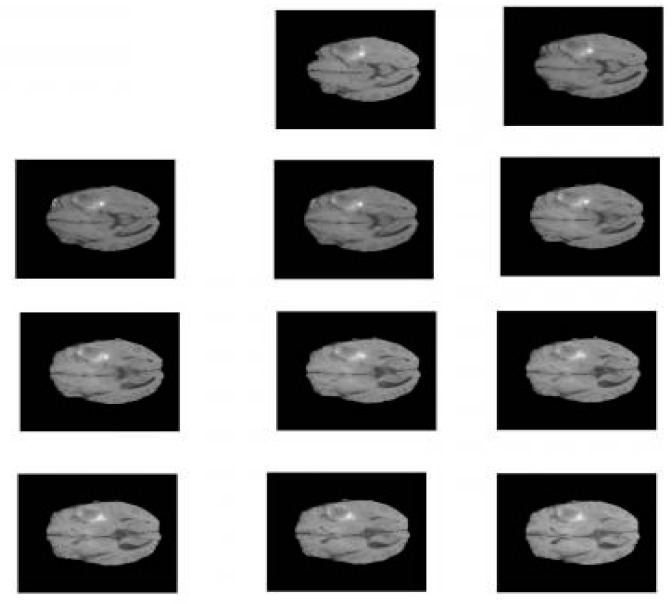
Sample images from BraTS.

BraTS 19 has been utilized for brain MRI images with a focus on the segmentations of brain tumors on multi-model magnetic resonance imaging (MRI) scans. BraTS contains MRI.jpg format images of 240 × 240. It contains 3762 brain MRI images of both males and females (see Figure 3) [3].

#### 3.2.2. OASIS

**Figure 4:**
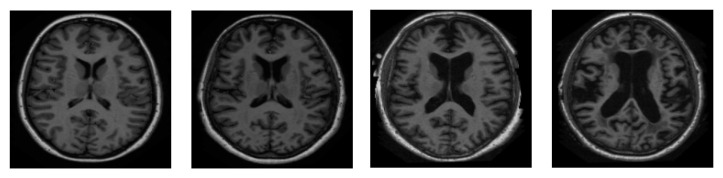
Sample images from OASIS.

The OASIS dataset (Open Access Series of Imaging Studies) has been utilized in this database for data analysis and the development of segmentation algorithms. It contains both male and female brain MRI images. It consists of 150 subjects aged 60 to 96 years. Each subject has been scanned on two or more visits, for a total of 373 imaging sessions. An OASIS project sample appears in Figure 4 [23].

#### 3.2.3. The Figshare

The Figshare dataset has been utilized as a database to analyze data and develop segmentation. It contains 3064 T1-weighted human brain MRI images of 233 patients. This dataset contains data organized in MATLAB R2021a (.mat files). Each file contains the fields of patient ID and image data. The Figshare information base appears in .mat format [24].

#### 3.2.4. The Kaggle Database

Kaggle, a subsidiary of Google L.L.C. (Mountain View, CA, USA), is an online community of machine learning and data science practitioners. Kaggle allows individuals to explore and publish datasets, explore and develop the models, associate with other machine learning and data science engineers in a web-based data science framework, and announce competitions to resolve data science obstacles. Kaggle launched in 2010 and now offers a collaborative data network, a workbench for cloud-based data science, and artificial intelligence education. The key employees are Anthony Goldbloom and Jeremy Howard. Nicholas Gruen succeeded Max Levchin as its founding chair. Equity was established in 2011, and the company was valued at about USD 25 million. On 8 March 2017, Google revealed that they had acquired datasets from Kaggle [25]. Images from all the datasets are further divided into training and test datasets, as shown in Table 1.

### 3.3. Preprocessing

**Figure 5:**
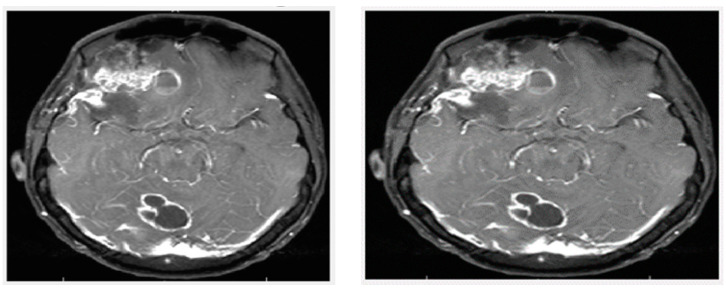
MRI image after preprocessing: (**Left**) Original image and (**Right**) Enhanced image.

In the preprocessing step, image enhancement is used as the first step. In this study, a contrast stretching filter was used for the enhancement of an MRI image for this greyscale image, since it functions relatively better. Without distorting the relative grey level intensities, the contrast stretching filters improved the contrast of an image [12]. As a consequence, an enhanced image does not offer an unnatural look like the equalization of histograms. Via contrast stretching, the image contrast is enhanced by stretching the spectrum of contrast values to spam the target scale from 0’s to 1’s. The uncertainty which can occur in the regions of images has been removed by contrast stretching enhancement. For this analysis, Figure 5 demonstrates the performance of a contrast-stretching-based enhancement test image.

To increase the sharpness and smoothness as well as for edge enhancement, a filter operation has been used for the processing of the image. In this proposed method, the anisotropic filter has been applied to the image instead of any other filter such as the Gaussian filter median and mean filter. The reason behind this is that while noise can be reduced better by using a mean filter, the edge cannot be preserved. On the other hand, the Gaussian filter makes the image blurry [13] while preserving the edge. The median filter is the most effective as compared to the Gaussian and mean filters, but it makes the image blurry. On the other hand, an anisotropic filter removes noise at a satisfactory level as well as preserving the edges. Moreover, in comparison to the Gaussian filter and median filter, an anisotropic filter makes an image less blurry.

The ultimate purpose of scanning an image is to eliminate digital image noise. The accuracy of the image is severely affected by the sounds. In a frame, there are several ways of getting rid of the noise, which is just about all the processing of the image. In a noisy environment, algorithms would not work properly. Therefore, as a pre-processing technique, image filters are used. For denoising purposes, an anisotropic filter is used among various filters in this study. To explain image diffusion, the generalized anisotropic diffusion equation is implemented.

The generalized anisotropic equation of diffusion is used to define the process of image diffusion; it is implemented as follows:

(1) $$\frac{ax}{at}=div\left(c\left(m,n,t\right)\vee 1\right)=\vee c.\vee x+c\left(m,n,t\right)\vee x\;$$

where ∨x is used for the representation of gradients of an image, and the $c\left(m,n,t\right)$ is used to describe the coefficient of the diffusion. A dimensionless estimation of the forward and reversed variations displays the following terminology:

(2) $$l_{i,j}^{1+i}=l_{i,j}^{t}\sum \left(k,I\right)\in N_{9}g\left(l_{k,l}^{t}-l_{i,j}^{t}\right)*\left(l_{k,l}^{t}-l_{i,j}^{t}\right)$$

(3) $$h\left(l_{k,l}^{t}-l_{i,j}^{t}\right)=\frac{c_{k,l}^{t}+c_{i,j}^{t}}{2}$$

Here, the 4th sides neighborhoods of the pixel’s It,j origin is presented by N4 = {(*i −* 1, *j*), (*i + 1*, j), (*i*, *j −* 1), (*i*, *j +* 1)}.

Out of notation, it seems that there is a heavy diffusion effect on the noise pixel and a weak diffusion effect on the signal pixel. Noise may then be eliminated and the signal preserved. For every iteration and the entire optimization method of the image, there are several designs of diffusion to follow the constant phase scale. A better iteration stage in Equations is suggested here [26].

(4) $$dt=\frac{1}{4c}$$

Herein, 1/4 is being used to guarantee the notation (35) would accumulate. The iterative method acquires the final feature step image [27]. Iteration error (*IE*) has been used to monitor a recursive number for the iterative procedure and its expression is:

(5) $$IE=\frac{\left\|l^{n}-l^{n-1}\right\|}{\left\|l^{n}\right\|}\leq T_{ie}$$

**Figure 6:**
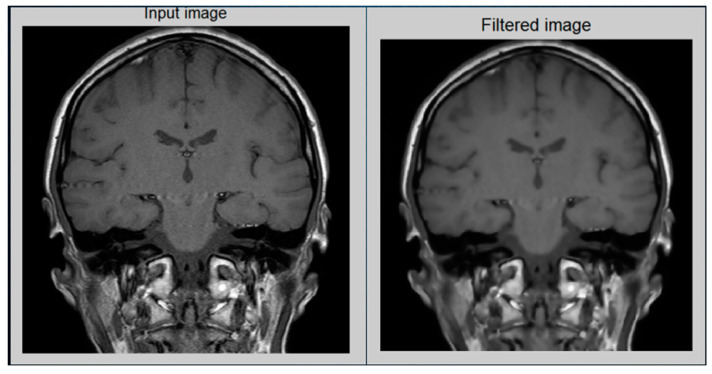
(**Left**): original image; (**right**): filtered image.

The recursive operation is completed when *IE* would be less than the equivalent for tolerance Tie. For this proposed approach, the test image is in Figure 6, which indicates the results.

Based on similar attributes, segmentation is used to segment a filtered image in its region. Basically, for further analysis, it is important to retrieve the vital characteristics from an image. The SVM method is used for thresholding due to its wide uses of segmentation on an image for further processing, such as the binary transformation of an image, as well as to analyze the features. The mechanism of propagation of an image into various components is known as the segmentation of the image. This is usually used in digital images to classify objects or other related information. Support vector machine (SVM) is used here among all the segmentation methods.

### 3.4. Feature Extraction

This is a process in which a higher level of information such as color, contrast, texture, and shape about an image is collected. For machine learning and human visual perception, texture analysis of an image is an important parameter. By selecting the prominent features of an image, texture analysis is efficiently used to boost the performance of the diagnostics device. Texture feature and the grey level of the co-occurrence matrix GLCM are one of the most widely used applications in image analysis that was introduced by Horalick et al. For feature extraction from medical images, this technique follows two steps. First, it computes the GLCM, while in the second step, the GLCM-based texture features are calculated. In the brain MRI images, it is an essential task to extract the relevant features from sophisticated diversification tissue compositions such as CSF, GM, as well as WM. Diagnosis, tumor staging, and the assessment of therapy response could be improved by the analysis and the texture findings of an image. The valuable statistical interface formulas are just as described for all the other critical features.

By adding the attributes of all the pixels, their intensities are separated by the cumulative variety of image pixels. An image average is measured as [2]

(6) $$M=\left(\frac{1}{m}*n\right)\overset{m_{1}}{\underset{x=0}{\sum }}\overset{n_{1}}{\underset{y=0}{\sum }}f\left(x.y\right)$$

The second actual key point is the standard deviation (*S.D*) that describes the probability distribution of the population that has been observed, and it may operate as the measurement of an inhomogeneousness. A greater value of standard deviation demonstrates the publicity of the high resolution of a boundary of an image as well as also indicating the better intensity level of an image [28]. It is calculated by the described formula:

(7) $$S.D\left(\sigma \right)=\sqrt{\left(\frac{1}{m*n}\right)\overset{m_{1}}{\underset{x=0}{\sum }}\overset{n_{1}}{\underset{y=0}{\sum }}{\left(f\left(x.y\right)-M\right)}^{2}}$$

An entropy has been calculated to summarize the nonlinearity of the compositional image, and it is represented [2] as shown in the equation below:

(8) $$\varepsilon =\sum_{x=0}^{m_{1}}\sum_{y=0}^{n_{1}}f\left(x.y\right)log2f\left(x.y\right)$$

Skewness is a measurement that has been used to measure a small asymmetry or the absence of symmetry [28]. For the *X* probability distribution, the skewness is represented as *Sk(x)* and it is described as follows:

(9) $$Sk\left(x\right)=\left\{\frac{1}{m}*n\right\}\frac{\sum \left\{F\left(x,y\right)-m\right\}3}{S.D^{3}}$$

Kurtosis is a parameter that is used to describe the shape of a random variable’s probability distributions [9]. The kurtosis for a random variable is described as the *Kurt* of variable *x* and it is represented as:

(10) $$Kurt\left(x\right)=\left\{\frac{.1}{m}*n\right\}\frac{\sum \left(F\left(x,y\right)-m\right)4|}{S.D^{4}}$$

The measurable value for the scope of iterations of the image pixels is described by a variable called energy. For the analysis of an image’s resemblance, a parameter called energy has been used. According to Horalicks, the GLCM’s features energy is defined. It should also be known as the second angular moment and is therefore identified as follows:

(11) $$En=\sqrt{\overset{m_{1}}{\underset{x=0}{\sum }}\overset{n_{1}}{\underset{y=0}{\sum }}F2\left(x.y\right)}$$

A measurement called by contrast (*Con*) is used over an image pixel’s resolution and its neighbors, and it has also been described as:

(12) $$Con=\overset{m_{1}}{\underset{x=0}{\sum }}\overset{n_{1}}{\underset{y=0}{\sum }}\left(x_{{\left(y\right)}^{2}f\left(x.y\right)}\right)$$

Then an image’s localized assimilation is determined by that of the inverse moments of differential (*I.D.M*). *I.D.M* can provide a specific number [29], or a set of values used to decide whether each image has a matte texture or not.

(13) $$I.D.M=\overset{m_{1}}{\underset{x=0}{\sum }}\overset{n_{1}}{\underset{y=0}{\sum }}\frac{1}{1+\left(x_{y}\right)2}f\left\{x.y\right\}$$

An image’s color and texture characteristics are defined throughout the directional moment (*D.M*) that is calculated by considering an alignment of an image as a measure in terms of an angle [30]. It is defined as:

(14) $$D.M=\overset{m-1}{\underset{x_{i}=0}{\sum }}\overset{n-1}{\underset{y_{i}=0}{\sum }}\{f\left(x_{i.}y_{i}\right)\left|x\_y\right|\}$$

Between all of these pixels, the repository in space is described by a feature called the correlation (*Corr*). It is defined as:

(15) $$Corr=\frac{\sum_{x_{i}=0}^{m_{1}}\sum_{y_{i}=0}^{n_{1}}\left\{\left(x.,y\right)f\left(x.,y\right)\right\}-MxMy}{horizontal\sigma x\sigma y}$$

Here, in the repositories in space horizontal’s domains, the mean and the standard deviations are represented by *Mx* and σx. While in the repositories in space vertical’s domains, the mean and standard deviations are represented by *My* and σy.

Within that textural evaluation of even an object, the resistivity is calculated by the measure that is called coarseness (*Cness*). A texture with a lower proportion would seem to be more rugged, in terms of elements of textures, than one with a large number, for a specified size of window frames [30]. If the texture is rough, it means it has a higher value of coarseness, while if the texture is fine, it means that it has a smaller coarseness value.

(16) $$Cness=\frac{1}{2^{m+n}}\overset{m-1}{\underset{x=0}{\sum }}\overset{n-1}{\underset{y=0}{\sum }}f\left(x,y\right)$$

To ensure better result analysis of the brain MRI images, apart from the extraction of this textural feature above, some parameters for the quality assessment are also needed that are described below.

Any image processing strategy other than by restriction or destruction of information in the communication of data is a cause of degradation in the quality of an image, and is signified by a perceptual matrix that is known as the structural similarity index matrix (*SSIM*) [31].

(17) $$SSIM=\left(\frac{\sigma_{xy}}{\sigma_{x}\sigma_{y}}\right)\left(\frac{2\bar{xy}}{\left(\bar{x^{2}}\right)+\left(\bar{y^{2}}\right)+C1}\right)\left(\frac{2\sigma x.\sigma y}{\left(\sigma x\right)2+\left(\sigma y\right)2+C2}\right)$$

If either *SSIM* value is strong, it ensures that the contrast, architectural substance, and brightness remain perfectly maintained.

An image fidelity or signal fidelity has a measure that is known as the mean square error. The objective of an image signal measurement of fidelity would be to figure out the fidelity or similarity among two different images, and this fidelity is determined by their provided quantitative score [31]. When the value of mean square error (*M.S.E*) is measured, it has also been considered therefore by some means whether a given image has been processed or distorted, whereas another one is the pristine original. This has been described as:

(18) $$M.S.E=\frac{1}{m}*n\sum \left\{f<x,y>-f<x,y>2\right\}$$

while the efficiency of restructuring a produced image is evaluated using a measurement named peak signal-to-noise ratio (*PSNR*). It is characterized as:

(19) $$PSBRindB=20{log}_{10}\left(\frac{2^{n}-1}{MSE}\right)$$

If mean square error (*M.S.E*) has a higher value, it means that it is indicating a better peak signal-to-noise ratio (*PSNR*).

Overlapping between the two different images is measured by the Dice coefficient and Dice similarity index. It has been described as:

(20) $$Dise\left(A.B\right)=\left\{2\times \frac{\left|A_{1}\wedge B_{1}\right|}{\left(\left|A_{1}\right|+\left|B_{1}\right|\right)}\right\}$$

### 3.5. Classification

Extracting a tumor region from an algorithm prediction originally suggested by Vladimir Vapnik and its updated incarnation contribute to the SVM original algorithm that was developed in 1993 by Vapnik and Cortes [32]. Such an approach was based on an analysis of the methodologies of deep classification and was intended to apply mostly to the N-class of binary classification and to a particular class of classification tasks. [33] The basic aim of these principles of SVM methodology is to convert all the discrete splitting objectives into an analog conversion by utilizing biomedical imaging from an international journal. The SVM technique has been utilized to transmit all discrete objectives into an analog transformation by utilizing a function that is known as SVM kernel functionality. In this study, we utilized a function for transformation that is a function of the Gaussian kernels. The functionality of the kernel is utilized to transform the nonlinear samples into a greater dimensional futures region where it might become possible to separate the data or non-linear samples by making the classification convention. While the expert ground truth is where B ε, here 0 indicates the minimum value of the Dice coefficient while 1 indicates the maximum value of the Dice coefficient. Between the two images, a higher value indicates a better overlap.

## 4. Experimental Results

See Table 1 for the distribution of the test and training datasets for each class representing the expression class.

The Tensorflow paradigm was used for the proposed method. The method’s efficiency was first assessed on the Kaggle dataset. The proposed model was trained on 253 examples and tested on 206 Kaggle images. Each image had dimensions of 225 × 225 × 3. Our proposed BTR system acquired 98 percent accuracy on the test set, and 98 percent accuracy on training the set. The outcome is shown in Table 2.

We used the BraTS dataset for assessment purposes as well. We trained the model with 1370 examples and 440 photos for research. The dimensions of every image were 256 × 256 × 3. Throughout our research trial, we used the same network setup. On BraTS, the total precision achieved was 99 percent. The outcome is shown in Table 3.

We have also used the OASIS dataset. A total of 560 images were chosen for training purposes and 559 images were chosen for testing purposes. Our BTR architecture has successfully achieved 98% accuracy. The result is shown in Table 4.

We have also used the Figshare dataset. A total of 80 images were chosen for training purposes and 73 images were chosen for testing purposes. Our BTR architecture has successfully achieved 99% accuracy. The result is shown in Table 5.

**Figure 7:**
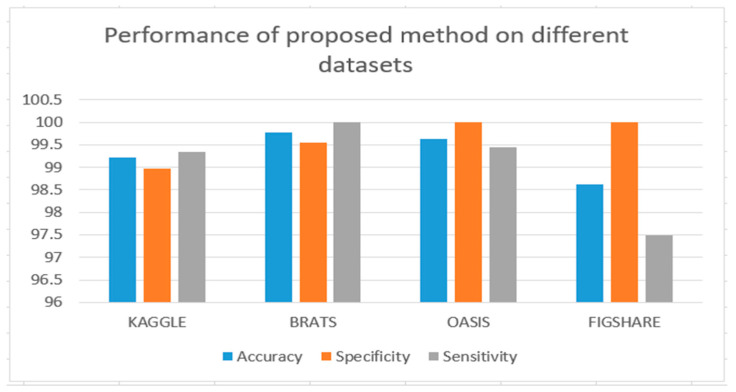
Performance of the proposed method on all datasets.

All databases’ performances are shown in Figure 7.

We have contrasted our framework with the experimental project’s state-of-the-art techniques. With limited training data, we obtained tremendous precision. Table 6 illustrates the performance report template.

## 5. Conclusions

In this study, we have proposed a machine learning approach for MRI-based brain tumor detection from DICOM datasets. Four datasets were used to evaluate the proposed approach properly using different evaluation measures. The method showed the best performance among state-of-the-art methods. It offers 98% accuracy as compared with other methods. We have used GLCM features to classify images after enhancing them using different preprocessing methods. In the future, we will use deep learning methods for further analysis and better performance of the model and the best detection rate for brain tumors.

## Figures and Tables

**Table 1:** Distribution of the set of images from each dataset for training and validation.

Type	Database Name	Abnormal	Normal	Total Images
Train Dataset	BraTS	1100	270	1370
	OASIS	560	559	1119
	Figshare	80	73	153
	Kaggle	1030	925	1955
Test Dataset	BraTS	120	120	440
	OASIS	560	559	1119
	Figshare	80	73	153
	Kaggle	155	98	253

**Table 2:** Confusion matrix for the Kaggle dataset using the BTR method.

	Abnormal	Normal
Abnormal	154	1
Normal	1	97

**Table 3:** Confusion matrix for the BraTS dataset using the BTR method.

	Abnormal	Normal
Abnormal	220	1
Normal	0	219

**Table 4:** Confusion matrix for the OASIS dataset using the BTR method.

	Abnormal	Normal
Abnormal	357	0
Normal	2	200

**Table 5:** Confusion matrix for the Figshare dataset using the BTR method.

	Abnormal	Normal
Abnormal	39	0
Normal	1	33

**Table 6:** Comparison with state-of-the-art methods.

Paper	Name of Model	Accuracy (%)
[32]	Support vector machine	92
[27]	CNN	97.8
[25]	Means fuzzy C means	97.5
[19]	Identification utilizing segmentation	97
Proposed Approach	Support vector machine	98

## Data Availability

All data available in the manuscript.
